# Probing Charge Generation Efficiency in Thin-Film Solar Cells by Integral-Mode Transient Charge Extraction

**DOI:** 10.1021/acsphotonics.1c01532

**Published:** 2022-03-31

**Authors:** Stefan Zeiske, Oskar J. Sandberg, Jona Kurpiers, Safa Shoaee, Paul Meredith, Ardalan Armin

**Affiliations:** † Sustainable Advanced Materials (Sêr-SAM), Department of Physics, Swansea University, Singleton Park, Swansea SA2 8PP, Wales, United Kingdom; ‡ Disordered Semiconductor Optoelectronics, Institute of Physics and Astronomy, University Potsdam, Karl-Liebknecht-Str. 24-25, 14476 Potsdam-Golm, Germany

**Keywords:** charge generation, thin-film solar cells, organic semiconductors, perovskite semiconductors, external generation efficiency

## Abstract

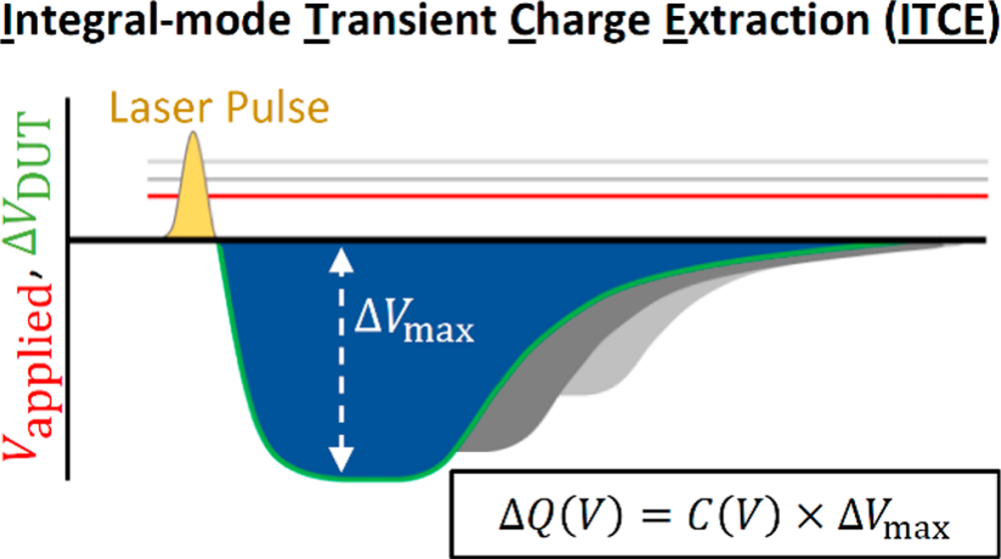

The photogeneration of free charges in light-harvesting devices is a multistep process, which can be challenging to probe due to the complexity of contributing energetic states and the competitive character of different driving mechanisms. In this contribution, we advance a technique, integral-mode transient charge extraction (ITCE), to probe these processes in thin-film solar cells. ITCE combines capacitance measurements with the integral-mode time-of-flight method in the low intensity regime of sandwich-type thin-film devices and allows for the sensitive determination of photogenerated charge-carrier densities. We verify the theoretical framework of our method by drift-diffusion simulations and demonstrate the applicability of ITCE to organic and perovskite semiconductor-based thin-film solar cells. Furthermore, we examine the field dependence of charge generation efficiency and find our ITCE results to be in excellent agreement with those obtained via time-delayed collection field measurements conducted on the same devices.

Organic semiconductors are characterized by incomplete free charge carrier generation at room temperature, which is directly related to their excitonic nature by a virtue of their low permittivity and thus incomplete screening of the electron–hole Coulomb force. To improve the charge generation efficiency, bulk heterojunctions (BHJ) comprising electron-donating (donor, D) and -accepting (acceptor, A) organic semiconductors are employed as the photoactive material in so-called BHJ organic solar cells (OSC). Free charge generation in these semiconductors ordinarily involves multiple steps starting with the photogeneration of singlet excitons in either the D or the A domains, followed by exciton diffusion to the D/A interface. At the D/A interface, excitons can undergo charge transfer (i.e., electron transfer from D to A or hole transfer from A to D) and form interfacial charge-transfer (CT) states,^1,2^ comprising Coulombically bound donor cations and acceptor anions. The charge transfer process (sometimes referred to as charge generation) is believed to be independent of any applied external electric field and predominantly energetically and kinetically driven.^3^ This mechanism can create photovoltage as the chemical potential of CT states becomes nonzero after charge generation,^4^ but it does not necessarily result in a considerable photocurrent. Efficient generation of free charge carriers (essential for photocurrent) requires CT states to quickly dissociate to free charges before decaying back to the ground state.^5−7^

However, the mechanism of CT state dissociation into free charges is still a matter of debate despite intensive studies over several decades. While the work of Braun^8^ implied that CT dissociation in OSCs is field-dependent, most efficient D/A blends show either no or only weak dependence on the electric field.^9−12^ Hence, more advanced models have been proposed to explain the fast and efficient dissociation of CT states to free charges. Clarke and Durrant, for instance, considered the role of entropy in CT dissociation events,^6^ while other models include the role of energetic disorder,^13^ delocalization,^14,15^ and vibronically excited (i.e., “hot”) states^16^ in the formation of free, separated charges. The role of “hot CT states” was challenged by Kurpiers and co-workers, who found the electric field and temperature dependent charge generation in fullerene acceptor (FA)-based BHJs to be independent of excess energy.^12^ They concluded, in line with past findings by Vandewal et al.,^2^ that charge generation proceeds through thermalized CT states, independent of activation energies and the energetic offset between relaxed singlet exciton and CT states. This is also to be expected in the new class of state-of-the-art OSCs based on nonfullerene acceptors (NFA) exhibiting low energetic offsets. Despite this, recent studies on CT dissociation conducted on NFA systems suggested an electric field and excess energy dependent charge generation.^17^ Furthermore, Karuthedath and co-workers proposed a model based on interfacial D/A band-bending inducing quadrupole moments, suggesting the requirement for an ionization energy offset to drive charge generation in both FA- and NFA-based OSCs.^18,19^

To gain more insight into the process of CT state dissociation, methods capable of probing free charge generation efficiency in thin-film solar cells independent of bulk recombination are needed. This has proven to be challenging but, if successful, could guide a better understanding of the mechanism of charge generation in state-of-the-art OSCs and thus aid molecular and architecture improvements. In the past, several measurement techniques have been employed to investigate free charge generation in optoelectronic devices. While intensity dependent photocurrent (IPC)^20^ and external (internal) quantum efficiency [EQE (IQE)]^21−42^ are prominent examples of steady-state techniques, transient absorption spectroscopy (TAS)^23−25^ and time-delayed collection field (TDCF) are, in turn, commonly used time-resolved techniques. Probing charge generation using IPC is questionable, as the results can be affected by first-order losses due to trap-assisted recombination and the so-called pseudo-first-order recombination near the electrodes.^26,27^ TAS, in turn, has been used to probe free charge generation via detecting geminate recombination at early time scales.^20,28^ However, TAS measurements are often performed in the transmission mode on thin films and not on fully optimized solar cell devices containing reflective back-electrodes. TDCF has been the most useful method and is frequently used to study the free charge generation dynamics in organic and perovskite solar cells.^12,29,30^ However, while TDCF remains a powerful methodology, it uses a complex circuit requiring specialist current preamplifiers with fast bias ramp-up times and suffers from *RC*-time limitations at short time scales.

In this work we advance an alternative and potentially more straightforward measurement technique to probe charge generation in optoelectronic devices. The technique is based on an extension of the integral-mode time-of-flight method^31^ in the low-intensity regime, which accounts for capacitive effects associated with the sandwich-type thin-film device structure. In contrast to TDCF, the proposed method does not suffer from limitations induced by *RC* effects, allows for a sensitive measurement of charge carrier density at very low pulse fluence without a reduced signal accuracy, and does not require ultrasensitive fast preamplifiers. The new method, however, has a more limited voltage range than TDCF. The analytical framework behind the technique, integral-mode transient charge extraction (ITCE), is derived and verified by drift-diffusion (DD) simulations. Finally, to demonstrate the method, we apply the technique to thin-film organic semiconductor and perovskite semiconductor (as a second verifying system) solar cells and probe the field-dependent external generation efficiency (EGE), finding good agreement of experimental results obtained via ITCE and TDCF conducted on the same devices.

## Methods and Materials

All devices were fabricated on ITO-patterned glass substrates (Lumtec). After cleaning the ITO substrates in DI water, acetone, and isopropanol, substrates were first dried by a nitrogen flow and then treated with a plasma for 1 min. Subsequently, 30 nm layers of PEDOT:PSS (Clevios PVP AI 4083) were spin-coated on substrates at 6000 rpm for 40 s, followed by thermal annealing under an inert atmosphere at 150 °C for 15 min. For PCDTBT:PC_70_BM active layers, PCDTBT (*M*_n_ = 65–85 kDa; purchased from Solaris Chem. Inc.54) and PC_70_BM (phenyl-C71-butyric acid methyl ester; purchased from Solenne BV) were mixed in dichlorobenzene at a concentration of 35 mg/mL with a donor/acceptor ratio of 1:4 (wt) and spin-coated at 2000 rpm to form a 100 nm thick film. For the neat PCDTBT active layer, 20 mg/mL PCDTBT was dissolved in chlorobenzene and spin-coated at 2000 rpm to form a 100 nm thick film. Triple cation perovskite active layers with a thickness of approximately 300 nm were prepared according to ref (32) using 10 nm PTAA as a hole-transport layer and 30 nm C_60_ and 7 nm LiF as an electron-transport layer. PCDTBT:PC_70_BM and neat PCDTBT (perovskite) devices were finalized by evaporating 7 nm Ca and 100 nm Al (8 nm BCP and 100 nm copper) through a shadow mask defining a pixel area of 0.16 cm^2^. Afterward, all devices were sealed with a cover glass using UV light-annealed glue (Bluefix).

A Newport Oriel Sol2A simulator in combination with a Keithley 2400 source-measure unit was used for current density versus applied voltage (*J–V*) characterization. A KG3 filtered reference silicon cell (calibrated at the Fraunhofer ISE) was used to calibrate the solar simulator to the standard AM 1.5G condition (100 mW cm^–2^).

**Figure 1:**
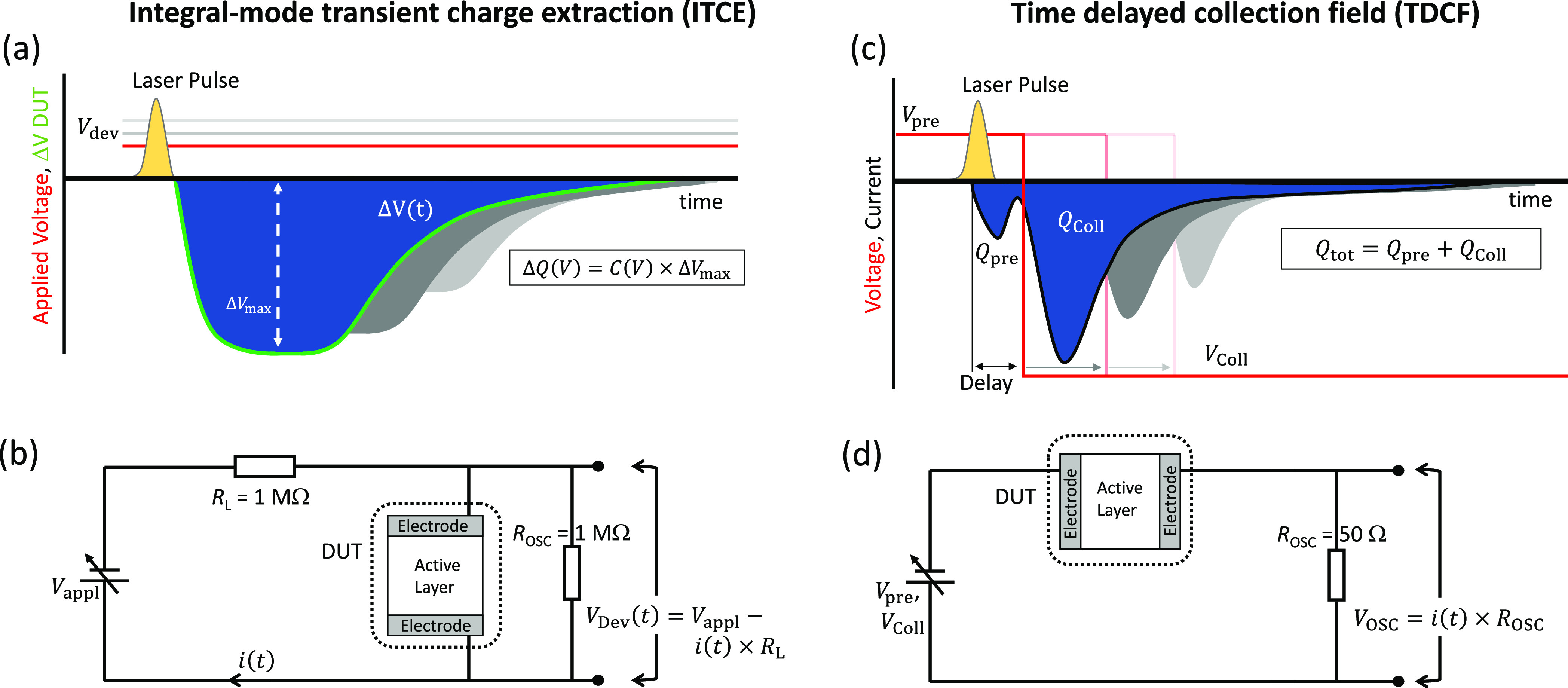
(a) Schematic timeline of an integral-mode transient charge extraction (ITCE) experiment. While the bias *V*_dev_ is applied on the DUT, a short laser pulse at *t* = 0 photogenerates charge carriers in the DUT active layer. The photoinduced change in voltage drop acorss the DUT active layer is measured by an oscilloscope in parallel with the DUT. The green (red) solid line indicates the corresponding photovoltage transient (applied device bias *V*_dev_). (b) Circuit of an ITCE experiment. A large load resistance *R*_L_ is in series with the DUT, while the change in photoinduced voltage drop across the DUT is measured by an oscilloscope with large input resistance configured in parallel. (c) Schematic timeline of a time delayed collection field (TDCF) experiment. At the time *t* = 0 a short laser pulse photogenerates charge carriers in the acitve layer of the DUT, while it is held under a prebias *V*_pre_. After a short delay time, a high reverse collection bias *V*_coll_ is applied on the DUT to extract all photogenerated charge carriers. The red (black) solid line indicates the correpsonding applied voltage (photocurrent) transient. (d) Simplified circuit of a TDCF experiment, where the DUT is in series with an oscilloscope with *R*_OSC_ = 50 Ω input impedance.

The schematic and circuit diagram of our ITCE method are shown in Figure 1a,b. Similar to the integral-mode time-of-flight method,^31^ a large load resistor and an external voltage source (to provide an external voltage *V*_appl_ to the circuit) are connected in series with the device under test (DUT). However, to record the voltage across the device, an oscilloscope is configured in parallel to the DUT. A short laser pulse is used to generate charge carriers in the bulk of the DUT. A diode-pumped, Q-switched Nd:YAG laser (Quantel, Viron Version A) operating a 532 nm excitation wavelength, 6.84 ns pulse width, 0.04 μJ cm^–2^ pulse fluence, and 20 Hz repetition rate is used in combination with a Standa 10MVAA attenuator to generate charge carriers in the bulk of the DUT. A Keithley 2450 is used to apply voltages across the DUT, which is in series with a 1 MΩ load resistor. The voltage transients are recorded with an oscilloscope (Rohde and Schwarz, RTM 3004) with 1 MΩ input resistance in parallel with the DUT. For dark *C–V* measurements, an E5061B ENA Network Analyzer with modulation frequency of 1 kHz and a bandwidth of 10 Hz is used. The voltage drop across the DUT is measured by a Keithley 2450.

Figure 1c,d schematically shows a simplified circuit and triggering diagrams of a typical TDCF experimental setup. Here, a variable prebias *V*_pre_ is applied on the operational photovoltaic DUT using an external voltage source, while a short laser photopulse leads to the generation of charge carriers in the photoactive layer. After a certain delay time *t*_delay_, the photogenerated charges are extracted by applying a collection bias *V*_coll_ (typically a high reverse bias). An oscilloscope is used to record the current flowing through the DUT, and by integrating the extraction photocurrent transient, the total number of extracted charge carriers can be obtained. More details of the TDCF setup are provided elsewhere.^33^

## Theory

ITCE is based on connecting the sandwich-type thin-film diode or solar cell device in series with a large load resistance *R*_L_ and a voltage source applying a DC bias *V*_appl_. The device is initially kept under DC conditions, with the corresponding voltage drop across the device being given by *V*_dev_ = *V*_appl_ – *i*_0_*R*_L_, where *i*_0_ is the DC current through the circuit. At the time *t* = 0, a light pulse is applied to the device, resulting in charge carriers being generated inside the active layer. The photogenerated electrons and holes are subsequently transported under the influence of the internal electric field toward the cathode and anode, respectively, giving rise to a transient current *i*(*t*) and a voltage drop *V*(*t*) = *V*_appl_ – *i*(*t*)*R*_L_ across the device.

In general, with the anode assumed to be located at *x* = 0 and the cathode at *x* = *d* (*d* is the active layer thickness), the corresponding time-dependent current density *j*(*t*) = *i*(*t*)/*A* (where *A* is the device area) is independent of the position *x* in the device and given by^34^
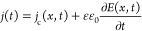
1Here, *E*(*x*,*t*) is the electric field and *j*_c_(*x*,*t*) is the conduction current density given by the sum of the individual electron and hole current densities, which both on the other hand depend on the position *x* in the active layer and the time *t*; ε is the relative permittivity and ε_0_ is the permittivity of the vacuum. Furthermore, the photoinduced change in the voltage drop Δ*V*(*t*) = *V*(*t*) – *V*_dev_ is related to the change of the electric field within the active layer via
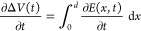
2Subsequently, upon taking the spatial average over the active layer of the total current in eq 1 and making use of eq 2, we obtain
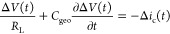
3where Δ*i*_c_(*t*) = (*A*/*d*)∫_0_^*d*^*j*_c_(*x*,*t*) d*x* – *i*_0_ is the change in the spatially averaged conduction currents induced by the light pulse (note that Δ*i*_c_(*t*) = 0 for *t* < 0), while  is the geometrical capacitance of the active layer.

For large load resistances (*R*_L_*C*_geo_ → ∞), eq 3 simplifies to ∂Δ*V*(*t*)/*∂t* = −Δ*i*_c_(*t*)/*C*_geo_. Under these conditions, the maximal induced change in the voltage is given as Δ*V*_max_ = Δ*Q*/*C*_geo_, where Δ*Q* = −∫_0_^*t*_extr_^Δ*i*_c_(*t*) d*t* is the total charge induced by the light pulse, while *t*_extr_ is the time taken for all photogenerated charge carriers to be extracted at the electrodes. After accounting for nonuniform charge distributions, it can be shown that Δ*Q* is related to the charge carrier densities inside the active layer via^35−37^
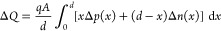
4assuming negligible charge carrier recombination (i.e., low intensity condition) and no trapping during the extraction process (0 < *t* ≤ *t*_extr_). Here, Δ*p*(*x*) = *p*(*x*,0) – *p*(*x*,*t*_extr_) and Δ*n*(*x*) = *n*(*x*,0) – *n*(*x*,*t*_extr_), where *p*(*x*,*t*) [*n*(*x*,*t*)] is the hole [electron] density within the active layer at position *x* and time *t*.

In general, Δ*p*(*x*) and Δ*n*(*x*) can be expressed as Δ*p*(*x*) = *n*_ph_(*x*) + Δ*p*_0_(*x*) and Δ*n*(*x*) = *n*_ph_(*x*) + Δ*n*_0_(*x*), where *n*_ph_(*x*) is the initial photogenerated carrier density at *t* = 0 and Δ*p*_0_(*x*) [Δ*n*_0_(*x*)] is the related induced change in the dark background hole [electron] density inside the active layer. In the case of an undoped device with noninjecting contacts, the background densities are negligibly small, and the active layer may be treated as an insulator; for this simplified case, eq 4 reduces to Δ*Q* = *C*_geo_Δ*V*_max_ = *qn̅*_ph_*Ad*, where *n̅*_ph_ ≡ (1/*d*)∫_0_^*d*^*n*_ph_(*x*) d*x* is the spatial average of the photogenerated carrier density at *t* = 0. However, most OSCs employ ohmic contacts. In these devices there exists a nonzero dark background density of electrons and holes, diffused from the contacts, accumulating near the cathode and anode contact, respectively.^37^ These dark charge distributions near the contacts effectively reduce the thickness of the insulator-like region in the active layer, resulting in an increased device capacitance relative to *C*_geo_.

Accounting for the presence of dark charge carriers, eq 4 can be expressed as Δ*Q* = *qn̅*_ph_*Ad* – Δ*Q*_0_. Here,  represents the corresponding charge induced by the difference between the background charge density profiles between *t* = 0 and *t* = *t*_extr_. However, since the background charge carrier profiles are determined by the prevailing applied voltage and electric field distribution (in contrast to the photogenerated charge *qn̅*_ph_*Ad*), Δ*Q*_0_ is capacitive, associated with a redistribution of the background charge profiles induced by the voltage change Δ*V*_max_ across the device. For small voltage perturbations Δ*V*_max_, we thus expect Δ*Q*_0_ = (∂*Q*_0_/∂*V*)Δ*V*_max_. Provided that *t*_extr_ ≪ *R*_L_*C* (large *R*_L_), we then finally obtain
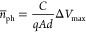
5where
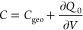
6is the voltage-dependent steady-state capacitance of the device in the dark at *V* = *V*_dev_. Hence, by measuring Δ*V*_max_ via ITCE as a function of the voltage *V*_dev_ across the device, in conjunction with dark device capacitance *C*, allows for *n̅*_ph_ versus *V*_dev_ to be calculated.

To verify the analytical treatment, we applied it to the result obtained from time-dependent DD simulations. The details of the DD model have been provided elsewhere.^37^ Briefly, in the simulations, we assumed a trap-free and undoped active layer with a thickness of 100 nm, a dielectric constant ε = 3, balanced mobilities of 10^–4^ cm^2^ V^–1^ s^–1^ for electrons and holes, and a bimolecular recombination coefficient of β = 5 × 10^–12^ cm^3^ s^–1^, corresponding to a Langevin reduction factor of ∼24. Further, a built-in voltage (*V*_bi_) of 1.2 V and ohmic contacts that are perfectly selective for the extraction of electrons and holes at the cathode and anode contact, respectively, were assumed. The device was specified to have an electrical area of *A* = 0.04 cm^2^ and connected in series with a large load resistance of *R*_L_ = 1 MΩ. The corresponding geometric capacitance of the device is *C*_geo_ ≈ 1.1 nF, amounting to an *RC* time of roughly 1 ms. Finally, the photogenerated carriers (introduced at *t* = 0) were taken to be generated with a uniform rate inside the active layer, with the corresponding density *n̅*_ph_ = *n*_ph_ assumed to be directly proportional to the pulse fluence. In this regard, geminate (first-order) recombination losses of excitons and charge-transfer states are assumed to be effectively included in *n*_ph_. To better demonstrate the capacitive effect, *n*_ph_ was assumed to be independent of the electric field in the simulations.

**Figure 2:**
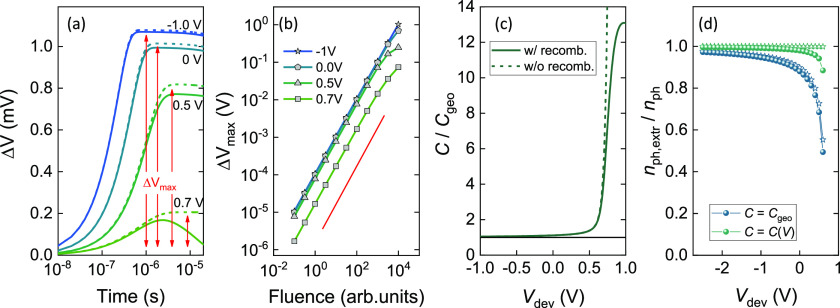
(a) Simulated voltage transients for different applied device voltages *V*_dev_ and compared for the cases with (solid lines) and without (dashed lines) recombination of charge carriers. (b) Voltage transient maxima, Δ*V*_max_, as obtained from the simulated voltage transients, plotted as a function of laser pulse fluence. The red solid line is a guide to the eye with a slope of 1. (c) Simulated device capacitance plotted as a function of applied voltage. The capacitance is normalized to the geometrical device capacitance *C*_geo_ (horizontal black line). The case with (without) recombination is indicated by solid (dashed) lines. (d) The extracted charge carrier density (*n*_ph,extr_), normalized to the generated carrier density (*n*_ph_), as obtained from the simulated voltage transients, and plotted as a function of device voltage *V*_dev_. Sphere-shaped (star-shaped) symbols correspond to the case with (without) recombination of charge carriers.

Figure 2a shows the simulated voltage transients (solid lines) for different *V*_dev_ ranging between −1 V and 0.7 V. The corresponding Δ*V*_max_ are plotted as a function of pulse fluence for different *V*_dev_ in Figure 2b. In Figure 2c, on the other hand, the device capacitance *C* under steady-state conditions in the dark (corresponding to low frequencies) is simulated as a function of *V*_dev_. In general, it can be seen that Δ*V*_max_ follows a linear dependence with the fluence at small Δ*V*_max_. At large enough fluences, however, Δ*V*_max_ eventually deviates from linearity as both higher order recombination and screening of the prevailing electric field start to play a role (as Δ*V*_max_ becomes comparable to *V*_dev_). On the other hand, Δ*V*_max_ is seen to strongly depend on *V*_dev_ at low fluences. We note that this dependence is present even for the idealized case when no recombination of charge carriers is present (β = 0, dashed lines). Instead, the *V*_dev_ dependence of Δ*V*_max_ is a consequence of the associated induced redistribution of the dark background charge carrier profile inside the active layer. As *V*_dev_ is increased, the diffusion of injected dark charges (from the electrodes) penetrates deeper into the bulk, effectively reducing the thickness of the neutral (insulator-like) region inside the active layer, manifest as an increased device capacitance relative to the geometrical capacitance *C*_geo_ (cf. eq 6). Figure 2d shows the extracted charge carrier density *n*_ph,extr_, as obtained from the simulations using eq 5, relative to the input photogenerated carrier density *n*_ph_. Indeed, *n*_ph,extr_ is closely given by *n*_ph_ when the device capacitance *C*(*V*) (Figure 2c) is used in eq 5. In contrast, if *C* = *C*_geo_ is assumed instead, a deviation between *n*_ph,extr_ and *n*_ph_ is observed, resulting in an underestimation of the photogenerated carrier density by a factor of *C*/*C*_geo_. In devices with ohmic contacts (Figure 2c), this underestimation becomes strongly dependent on the voltage in the forward bias and may be mistaken as an apparent field dependence of EGE; hence, to correctly obtain *n*_ph_, the voltage dependence of the device capacitance must be accounted for.

We note that there is a small deviation taking place between *n*_ph,extr_/*n*_ph_ of the cases with and without recombination in the active layer at large *V*_dev_ approaching the built-in voltage; this deviation can be attributed to additional (pseudo)first-order recombination taking place between photogenerated charge carriers and dark background charge carriers near the electrodes.^26,38^ In principle, this additional loss may be minimized by tuning the optical electric field (e.g., careful choice of the laser wavelength or the introduction of optical spacer layer) such that the generation profile peaks in the middle of the active layer and is minimal near the electrodes. It should be stressed that, in the case of nonideal contacts, surface recombination (i.e., the collection of minority carriers at the “wrong” electrode) may become prevalent as well, presenting an additional voltage-dependent first-order recombination channel.^39^

From the above presented theoretical and numerical analyses, we conclude that photogenerated charge carrier densities in thin-film solar cells can be measured sensitively via ITCE, when (i) higher-order recombination processes are not present, and (ii) (voltage dependent) carrier back-injection and diffusion-mediated redistribution of dark background charges in the photoactive layer of the DUT are accounted for. While (i) can be addressed by recording ITCE voltage transients at low pulse fluence and avoiding too high Δ*V*_max_ (Δ*V*_max_ should be as small as possible, preferably well below 10 mV), (ii) can be addressed by accurately measuring the voltage-dependent device capacitance (at low enough frequencies) in the dark. In the following, we will implement those findings and probe the EGE in different thin-film organic semiconductor and perovskite semiconductor solar cells.

## Results and Discussion

We first applied ITCE to the well-understood model organic solar cell, PCDTBT:PC_70_BM, to further validate the theoretical/numerical findings. Furthermore, we examined neat PCDTBT photovoltaic cells, as well as a high efficiency triple cation perovskite thin-film solar cells. We studied the field dependent EGE in these systems via ITCE and compared these data with benchmark TDCF results. To this end, EGE is evaluated as a function of *V*_dev_, noting that the (DC) electric field is expected to be uniform and scale linearly as *E* = (*V*_dev_ – *V*_bi_)/*d*, with *V*_bi_ on the order of 1 V in these devices. This is expected to be a good approximation for thin active layers and voltages well below *V*_bi_.

**Figure 3:**
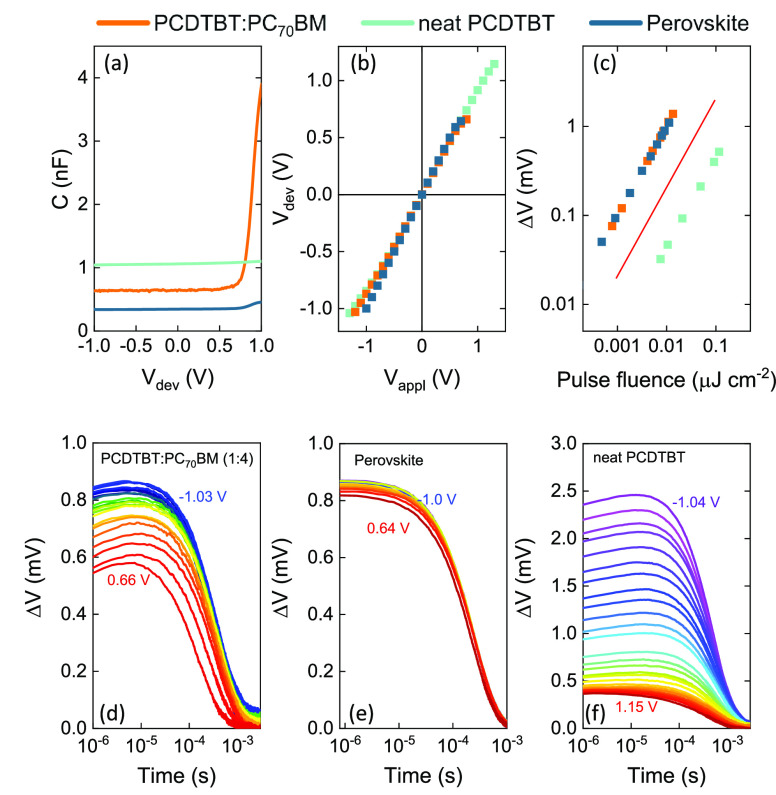
(a) Device capacitance in the dark plotted as a function of voltage and compared with PCDTBT:PC_70_BM (1:4), neat PCDTBT, and perovskite thin-film solar cells. A bandwidth of 10 Hz and modulation frequencies of 1 kHz (PCDTBT:PC_70_BM (1:4), neat PCDTBT) and 50 kHz (perovskite) were used. (b) Relation between applied circuit voltages (*V*_appl_) and the measured voltage drops (*V*_dev_) across the three devices. (c) Maximum change Δ*V*_max_, as obtained from voltage transients, for all three solar cells plotted as a function of laser pulse fluence. The excitation wavelength was set to λ_exc_ = 532 nm, and no bias voltage was applied on the devices (short-circuit). The red solid line is a guide to the eye with a slope of 1, indicating the absence of higher-order photocurrent loss mechanisms. (d) Voltage transients of a PCDTBT:PC_70_BM (1:4) thin-film solar cell compared for different applied bias voltages. (e) Repetition of panel (d), but plotted for a perovskite solar cell. (f) Repetition of panel (d), but plotted for a neat PCDTBT solar cell.

Figure 3a shows the dark capacitances of all three devices plotted as a function of device voltage, *V*_dev_. As shown, the PCDTBT:PC_70_BM and perovskite thin film solar cells show changes in device capacitance when *V*_dev_ approaches *V*_bi_. To account for the DC voltage loss across the load resistance, the relations between the applied circuit voltage *V*_appl_ and the measured voltage drop *V*_dev_ across the PCDTBT:PC_70_BM, neat PCDTBT, and perovskite thin-film devices are depicted in Figure 3b. On the other hand, Figure 3c shows the Δ*V*_max_ at short-circuit, as obtained from the voltage transients, plotted as a function of laser pulse fluence, and compared for all three thin-film solar cells. We took great care to avoid high laser pulse fluences (which induce substantial bimolecular recombination) when recording the voltage transients at different *V*_dev_. The red solid line in Figure 3c is a guide to the eye with a slope of 1, indicating the absence of higher-order (e.g., bimolecular) recombination processes. The corresponding ITCE voltage transients for the PCDTBT:PC_70_BM, neat PCDTBT, and perovskite solar cell are shown in Figure 3d–f, from which Δ*V*_max_ was obtained at the voltage plateaus.

**Figure 4:**
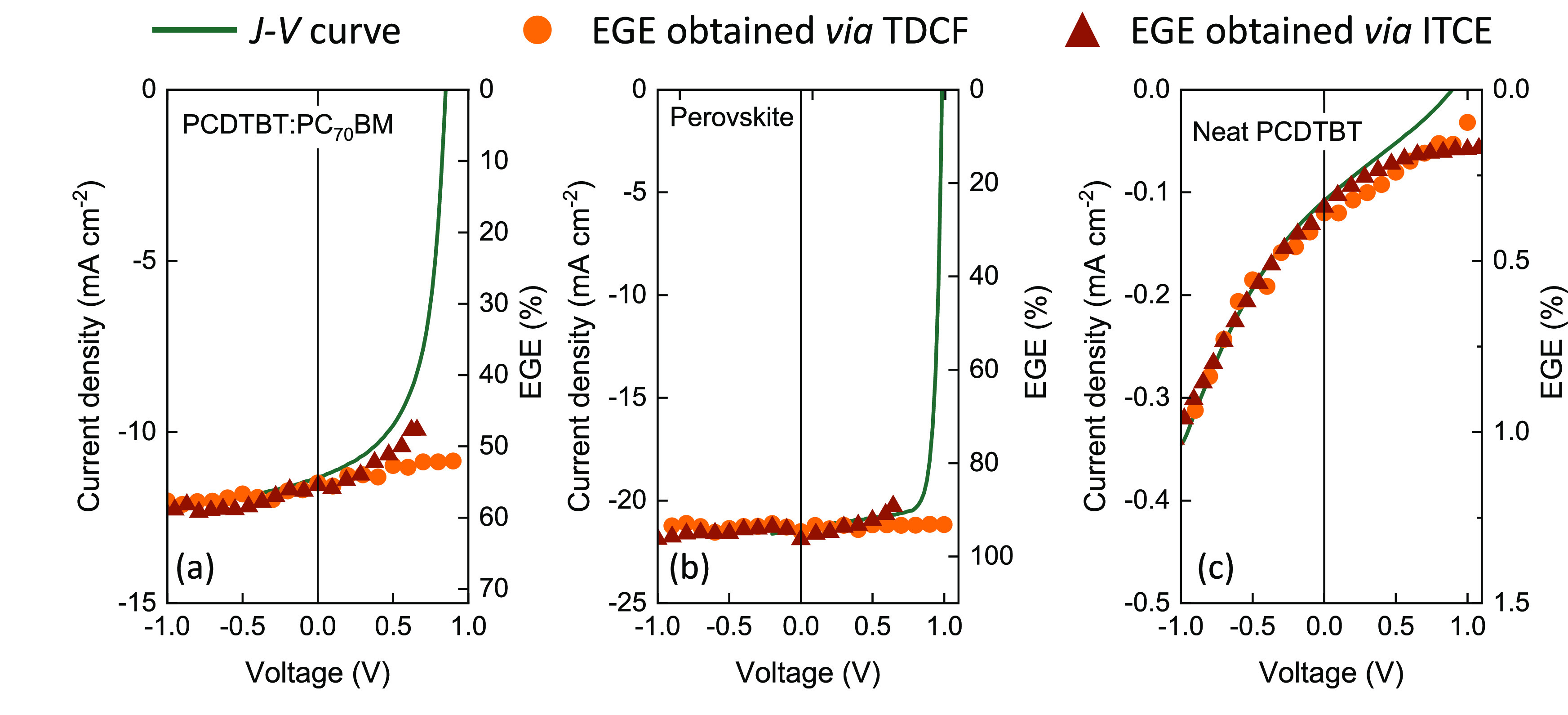
(a) *J*–*V* characteristics (solid line) of a thin-film PCDTBT:PC_70_BM (1:4) solar cell measured under artificial 1 sun (AM 1.5G) illumination and compared with the external generation efficiency (EGE) obtained via TDCF (orange symbols) and ITCE (red symbols). (b) Repetition of panel (a), but plotted for a thin-film perovskite solar cell. (c) Repetition of panel (a), but plotted for a neat PCDTBT device.

From the *C*–*V* curves and voltage transients we calculated the EGE, which was determined based on the photogenerated charge carrier density (*n*_ph_) and the pulse photon density (*N*_ph_) via EGE = *n*_ph_/*N*_ph_, where , λ is the laser pulse excitation wavelength, *h* is the Planck constant, and *F* denotes the pulse fluence (in the unit of J). The ITCE results were cross-calibrated with those obtained via TDCF conducted on the same devices. Figure 4a compares the *J*–*V* curve of the PCDTBT:PC_70_BM solar cell (solid line) with the EGE obtained via ITCE (red symbols) and TDCF (orange symbols). Our ITCE-based EGE results are in excellent agreement with those obtained via TDCF. We find the EGE in PCDTBT:PC_70_BM to show a weak field dependence decreasing slightly with increasing forward bias voltages. We note, however, that due to expected nonuniform electric fields and uncertainties in the measured device capacitance at high voltages (i.e., when *V*_dev_ approaches the built-in voltage), the trustable EGE regime in ITCE is limited to *V*_dev_ below ∼0.66 V in the forward bias direction. This is partly due to the rapid increase of the capacitance with voltage (see Figure 3a), where the value of *C* becomes more sensitive to small voltage fluctuations (Δ*V*_max_) and partly due to strong recombination and space charge effects affecting the measured capacitance at large bias.

In a similar manner, we investigated the EGE in a thin-film perovskite solar cell (see Figure 4b), where we find the EGE to be field-independent. Again, our ITCE results (red symbols) show good agreement with those obtained via TDCF. Similar to the PCDTBT:PC_70_BM device, the trustable *V*_dev_ window is, when probed by ITCE, limited to ∼0.64 V in forward bias direction. We note that perovskites are quite different to organic semiconductors in that they are predominantly nonexcitonic at room temperature and thus demonstrate a more general (if not universal) applicability of ITCE to thin-film photovoltaic devices.

Finally, we investigated a system with an electric field-dependent EGE. To this end, a neat PCDTBT thin-film device was used. It is well-established that single-component organic solar cells exhibit field dependent charge generation.^40,41^ Therefore, a neat PCDTBT device is an appropriate model system to observe the field dependence. We note that the capacitance of this device showed a weaker voltage dependence (see Figure 3a), allowing for the capacitance to be accurately measured over the entire voltage range. Subsequently, as shown in Figure 4c, the field-dependent EGE results obtained via ITCE (red symbols) and TDCF (orange symbols) are in excellent agreement over the entire bias voltage regime.

In contrast to the PCDTBT:PC_70_BM and perovskite devices, the accuracy of the neat PCDTBT *C*–*V* measurement at large forward bias voltages was not influenced by carrier diffusion and back-injection from the electrodes into the photoactive layer; this can mainly be attributed to the nonohmic injection character of one or both of the electrodes, suppressing strong recombination and space charge effects at large voltages. In this regard, it should be noted that the EGE is a property of the photoactive layer, hence a modification of the device stack aimed at a more precise *C*–*V* measurement (or, suppression of diffusion of injected dark charges, recombination, and the buildup of space charge) allows for accurate ITCE measurements over the entire voltage regime.

## Conclusions

We have presented a transient measurement technique, ITCE, to probe charge generation efficiency in thin-film solar cells, which is based on the sensitive measurement of pulsed, photoinduced changes in voltage drop across the active layer, combined with capacitance measurements. A simple series-circuit with large *RC*-time is used to generate voltage transients at low laser pulse fluence from which the maximum change in active layer voltage drop can be determined. We derived and verified the theoretical framework of ITCE by DD simulations and demonstrated its applicability by probing the field dependence of EGE in thin-film perovskite and organic solar cells. Our results are in good agreement with those obtained via TDCF conducted on the same devices.

Despite the limitations of ITCE at high forward bias voltages due to uncertainties in the accurate measurement of the device capacitance, ITCE operates at very low pulse fluence (avoiding higher-order recombination) and does not suffer from *RC*-time limitations. Hence, ITCE with its much simpler circuit allows the measurement of small charge carrier densities sensitively and can be used in a complementary manner with the more complex TDCF method to probe the field dependence of charge generation in thin film solar cells.
